# Morroniside Protects Human Granulosa Cells against H_2_O_2_-Induced Oxidative Damage by Regulating the Nrf2 and MAPK Signaling Pathways

**DOI:** 10.1155/2022/8099724

**Published:** 2022-09-09

**Authors:** Yucong Ma, Guimin Hao, Xiaohua Lin, Zhiming Zhao, Aimin Yang, Yucong Cao, Shuancheng Zhang, Lijie Fan, Jingran Geng, Yu Zhang, Jingwei Chen, Cuimiao Song, Ming He, Huilan Du

**Affiliations:** ^1^Hebei Key Laboratory of Integrative Medicine on Liver-Kidney Patterns, Institute of Integrative Medicine, College of Integrative Medicine, Hebei University of Chinese Medicine, Shijiazhuang, Hebei 050091, China; ^2^Department of Reproductive Medicine, The Second Hospital of Hebei Medical University, Shijiazhuang, Hebei 050000, China; ^3^Hebei Hospital of Traditional Chinese Medicine, Shijiazhuang, Hebei 050011, China; ^4^Department of Physiology, Basic Medical College, Hebei University of Chinese Medicine, Shijiazhuang 050091, China

## Abstract

Morroniside is the main ingredient of *Cornus officinalis* and has a variety of biological activities including antioxidative effects. Ovarian granulosa cells (GCs) are responsible for regulating the development and atresia of follicles, which are susceptible to oxidative stress. In this study, we determined whether morroniside can inhibit the oxidative stress of GCs induced by hydrogen peroxide (H_2_O_2_), leading to improved oocyte quality. The oxidative damage and apoptosis of ovarian GCs cultured *in vitro* were induced by the addition of H_2_O_2_. After pretreatment with morroniside, the levels of ROS, MDA, and 8-OHdG in ovarian GCs were significantly decreased. Morroniside significantly upregulated p-Nrf2 and promoted the nuclear translocation of Nrf2, which transcriptionally activated antioxidant SOD and NQO1. In addition, morroniside significantly regulated the levels of apoptosis-related proteins Bax, Bcl-2, cleaved caspase-9, and cleaved caspase-3 via the p38 and JNK pathways. These results suggest that morroniside can reduce the oxidative damage and apoptosis of ovarian GCs induced by H_2_O_2_.

## 1. Introduction

Ovarian granulosa cells (GCs) are located in the follicles and around the oocytes. They produce steroids, which are responsible for regulating the development and atresia of follicles. GCs are very important for oocyte maturation, oocyte quality, and embryo development [1, 2]. Adenosine triphosphate in ovarian GCs can be directly transferred to oocytes through the gap of cumulus GCs [3]. GCs can also convert glucose into pyruvate, the energy substrate of oocytes, and transfer it to oocytes [4]. The dysfunction of GCs is related to ovarian senescence, the fewer oocytes retrieved, poor oocyte and embryo quality, and low pregnancy rate of *in vitro* fertilization-embryo transfer (IVF-ET) [5, 6]. Additionally, women with polycystic ovary syndrome (PCOS) and endometriosis have a higher rate of GC apoptosis, thus reduced fertility and pregnancy rates [5, 7]. Therefore, normal ovarian GCs are necessary to maintain reproductive function.

Physiological levels of reactive oxygen species (ROS) are necessary for follicular growth, oocyte maturation, normal ovulation, and ovarian hormone synthesis [8]. Oxidative stress occurs with the generation of excessive ROS or when antioxidant defense mechanisms are weakened [9]. Oxidative stress is the basic pathogenesis of a variety of reproductive system diseases, which can damage fertility, decrease pregnancy and delivery rate, and result in recurrent abortion [10]. Eight-hydroxy-2′-deoxyguanosine (8-OHdG) is a sensitive indicator of DNA damage as the result of oxidative stress. The increase of 8-OHdG content in ovarian GCs is related to the low fertilization rate of oocytes and poor embryo quality during IVF-ET [11]. Nuclear factor erythroid 2-related factor 2 (Nrf2) is a key antioxidant transcription factors in response to ROS. It binds to the antioxidant response element (ARE) and induces the expression of numerous antioxidant enzymes including superoxide dismutase (SOD) [12, 13]. A high level of SOD is positively correlated with IVF pregnancy rate [14]. In addition, excessive ROS generation can trigger GCs apoptosis through mitogen-activated protein kinase (MAPK), protein kinase B (AKT), and mammalian target of rapamycin (mTOR) pathways and increased the expression of apoptosis-related genes expressions, including caspase-9 and caspase-3. These effects cause GC cycle arrest and reduce its supporting effect on oocytes, thereby affecting oocyte development, ovarian reserve, and reproductive potential [15, 16]. Therefore, there is an urgent need to identify drugs that can reduce oxidative stress in GCs, to improve female reproductive function.

**Figure 1:**
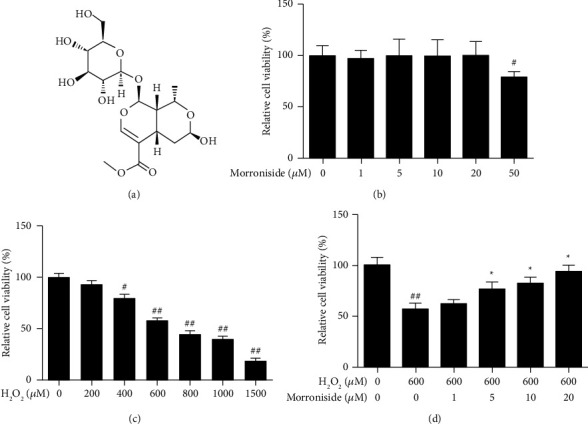
The effect of morroniside on GCs viability decreased by H_2_O_2_. (a) Structure of morroniside. (b) Ovarian GCs were treated with morroniside at different concentrations (including 1, 5, 10, 20, 50 *μ*M) for 24 h. (c) GCs were treated with H_2_O_2_ at different concentrations (including 200, 400, 600, 800, 1000, 1500 *μ*M) for 24 h. (d) GCs were pretreated with morroniside at different concentrations (including 5, 10, and 20 *μ*M) for 24 h and then treated with 600 *μ*M H_2_O_2_ for 24 h. The survival rates of GCs were determined by CCK-8 assay kit. Data represent mean ± SD, *n* = 6. ^#^*P* < 0.05 versus the control group, ^##^*P* < 0.01 versus the control group, ^*∗*^*P* < 0.05 versus the H_2_O_2_ group.

*Cornus officinalis* is among the most commonly used Chinese medical herbs, and morroniside (Figure 1(a)) is the most abundant iridoid glycoside in *C. officinalis* [17]. It has a variety of biological activities, such as antioxidant, antiapoptotic, and anti-inflammatory effects [18–20], which can relieve nerve pain and improve cardiovascular and liver functions [21, 22]. Deng et al. [23] showed that morroniside inhibited autophagy in rat ovarian GCs by regulating the phosphoinositide 3-kinase (PI3K)/AKT/mTOR pathway. However, the effects of morroniside on the oxidative stress of GCs induced by hydrogen peroxide (H_2_O_2_) is largely unclear.

Therefore, the aim of this study was to investigate the effect of morroniside on the oxidative stress of GCs induced by H_2_O_2_ and elucidated the molecular mechanisms by which morroniside protects human GCs against H_2_O_2_-induced oxidative damage which could improve oocyte development.

## 2. Materials and Methods

### 2.1. Cell Culture and Treatment

Ovarian GCs were obtained from patients undergoing a long-term gonadotropin-releasing hormone downregulation due to fallopian tube factors at the Reproductive Department of the Second Hospital of Hebei Medical University (Shijiazhuang, China). The study was approved by the ethics committee of the hospital, and patients provided written informed consent. Follicular fluid-containing GCs were centrifuged at 4°C (433 × *g*, 10 min). Then, the upper follicular fluid was removed, and 5 mL phosphate-buffered saline (PBS) was added to the lower sediment and mixed. Next, 5 mL human lymphocyte separation fluid (Lympholyte-H; Cedarlane Laboratories, Ontario, Canada) was added to another 10 mL centrifuge tube and inclined at an angle of 45°. PBS suspension was slowly added to the surface of the human lymphocyte separation solution and then centrifuged at 4°C (680 × *g*, 10 min). The white floc in the middle layer was the GCs. To avoid intergroup differences due to individual patient differences, we mixed GCs collected from all patients on that day, made a cell suspension, cultured the cells in a plate, and administered different intervention drugs.

GCs were cultured in DMEM/F12 medium (Gibco, Thermo Fisher Scientific, Waltham, MA, USA), supplemented with 10% (v/v) fetal bovine serum (Gibco, Thermo Fisher Scientific) and 1% (v/v) penicillin/streptomycin (Solarbio, Beijing, China) at 37°C in a 5% CO_2_ incubator. The medium was replaced after 24 hours. H_2_O_2_ (Sigma, St. Louis, MO, USA) and N-acetyl-cysteine (NAC, purity ≥98%; Solarbio) were diluted in PBS, and morroniside (purity = 98.55%; MedChemExpress (MCE), Shanghai, China) was dissolved in dimethyl sulfoxide to suitable concentrations. The GCs were pretreated for 24 h with different morroniside concentrations of 1, 5, 10, 20, and 50 *μ*M or NAC of 1 mM and 5 mM and then incubated with H_2_O_2_ for 24 h.

### 2.2. Lentivirus Vector

Nrf2 shRNA (GeneChem, China) was generated with GCTCGCATTGATCCGAGATAT (sh-Nrf2). A control vector was generated with the control oligonucleotide TTCTCCGAACGTGTCACGT.

### 2.3. Cell Counting Kit-8 Assay

GCs were pretreated with different concentrations of morroniside or H_2_O_2_, and the effects on cell viability were determined by the Cell Counting Kit-8 (CCK-8) assay (MCE). Briefly, 10 *μ*L CCK-8 reagent was added to cells in a 96-well plate and incubated at 37°C for 2 h. The optical density value of each well was measured at 450 nm with a microplate reader (VesarMax; Molecular Devices, Sunnyvale, CA, USA).

### 2.4. Intracellular ROS Detection

Intracellular ROS levels in each group were detected with the ROS Assay Kit (Beyotime, Shanghai, China). The culture medium containing serum was removed, and the cells were incubated with diluted DCFH-DA (1 : 1000) for 20 min at 37°C in a 5% CO_2_ incubator, followed by three washes with serum-free medium. ROS content was detected by fluorescence microscopy (EVOS® FL Cell Imaging System, Thermo Fisher Scientific), and fluorescence intensity was analyzed with ImageJ software.

### 2.5. ELISA Assay

The ovarian GC sample lysis fluid was diluted to the optimal concentration. The biomarkers content related to oxidative stress and apoptosis, including ROS, malondialdehyde (MDA), 8-OHdG, total antioxidant capacity (T-AOC), SOD, NAD(P)H quinone oxidoreductase 1 (NQO1), and caspase-3, were detected with an ELISA kit (Jianglai Biological Co., Ltd., Shanghai, China; Jiancheng Bioengineering Institute, Nanjing, China; Abcam, Cambridge, MA, USA; Tongwei Industrial Co., Ltd., Shanghai, China) according to the manufacturers' instructions. The absorbance values were measured with a microplate reader (VersaMax; Molecular Devices).

### 2.6. Western Blot Analysis

The collected GCs were lysed, and total and nuclear protein was extracted according to the instructions of a nuclear and cytoplasmic protein extraction kit (Beyotime). Total protein (10–15 *μ*g/well) was separated by 10% sodium dodecyl sulfate-polyacrylamide gel electrophoresis and electrotransferred to a polyvinylidene difluoride (PVDF) membrane (Millipore, Darmstadt, Germany). The PVDF membrane was blocked in 5% milk for 2 h and incubated overnight at 4°C with the following primary antibodies: SOD (1 : 1000, ab68155; Abcam, Cambridge, MA, USA), NQO1(1 : 1000, ab80588; Abcam), B-cell lymphoma 2 (Bcl-2) (1 : 1000, 26593-1-AP; Proteintech, Rosemont, IL, USA), Bcl-2-associated X protein (Bax) (1 : 1000, ab32503; Abcam), cleaved caspase-3 (1 : 1000, YM3431; ImmunoWay, Plano, TX, USA), cleaved caspase-9 (1 : 1000, YC0013; ImmunowWay), phosphorylated Nrf2 (p-Nrf2) (1 : 500, ab76026; Abcam), Nrf2 (1 : 500, ab62352; Abcam), phosphorylated extracellular signal-regulated kinase (p-ERK) (1 : 500, YP0101, ImmunoWay), ERK (1 : 500, 16443-1-AP; Proteintech), c-Jun N-terminal kinase (JNK) (1 : 500, 66210-1-lg; Proteintech), phosphorylated JNK (p-JNK) (1 : 500, YP0156; ImmunoWay), p38 (1 : 500, ab31828; Abcam), and phosphorylated p38 (p-p38) (1 : 500, ab4822; Abcam). The PVDF membrane was washed three times with Tris-buffered saline with 0.1% Tween 20 (TBST) and then incubated with the secondary antibody (SA00001-2; Proteintech) at room temperature for 1 h. After another three washes with TBST, antibody-antigen complexes were visualized using the Chemiluminescence Plus Western Immunoblot Analysis Kit (Millipore). The images were collected by a chemiluminescence imager (ImageQuant LAS 4000; GE Healthcare, Chicago, IL, USA) and quantitatively analyzed with ImageJ software.

### 2.7. Immunofluorescence Staining

The treated GCs were fixed in 4% paraformaldehyde for 20 min, permeabilized with 1% Triton, blocked in 10% goat serum for 30 min, and incubated overnight with Nrf2 antibody (1 : 200) at 4°C. Then, the cells were incubated with fluorescence-labeled secondary antibodies at room temperature for 2 h, followed by staining with DAPI for 10 min. Finally, cells were observed under a laser scanning confocal microscope (Leica, Wetzlar, Germany).

### 2.8. Statistical Analyses

All data are expressed as the mean ± standard deviation. Statistical analyses were performed using SPSS 21.0 software (SPSS Inc., Chicago, IL, USA). Comparisons were performed by one-way analysis of variance followed by post-hoc analysis. *P* < 0.05 was considered statistically significant.

## 3. Results

### 3.1. Morroniside Increases GC Viability

The CCK-8 assay was used to determine the viability of cells treated with different concentrations of morroniside or H_2_O_2_. Compared with the control group, there was no significant change in GC viability after pretreatment with 1, 5, 10, or 20 *μ*M morroniside (Figure 1(b)). With an increase in H_2_O_2_ concentration, the viability of GCs treated with H_2_O_2_ gradually decreased in a concentration-dependent manner; 600 *μ*M H_2_O_2_ (57.9 ± 2.7% of the control group) was chosen for subsequent experiments (Figure 1(c)). After preincubation with different concentrations of morroniside, the viability of H_2_O_2_-treated GCs was significantly increased compared to cells treated with H_2_O_2_ alone (Figure 1(d)).

### 3.2. Morroniside Inhibits GC Oxidative Stress

**Figure 2:**
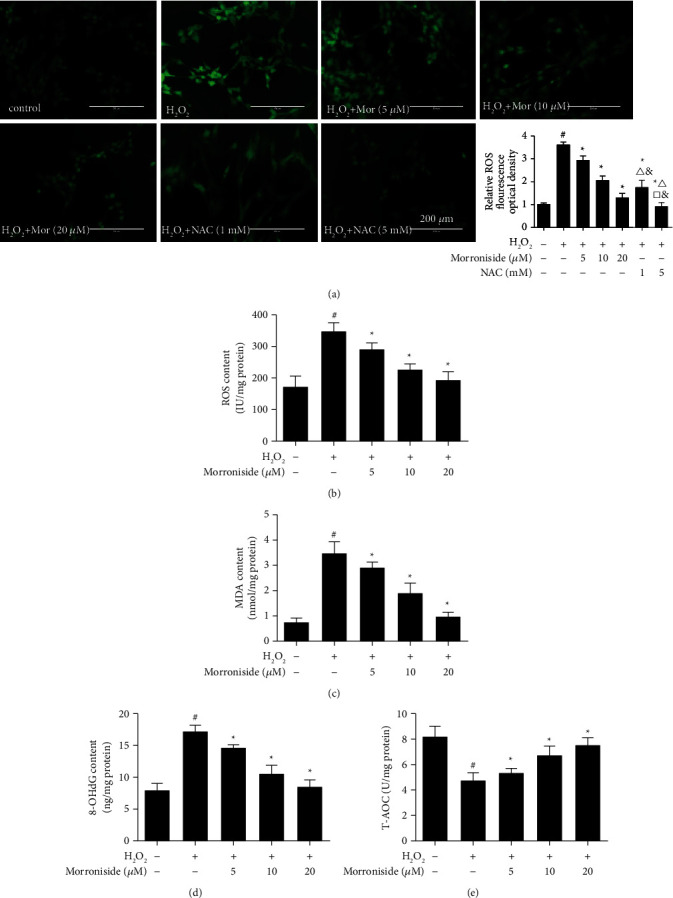
Morroniside inhibited GCs oxidative stress induced by H_2_O_2_. (a) Intracellular ROS level and quantitative analysis of ROS. Data represent mean ± SD, *n* = 3. The oxidative stress biomarkers of (b) ROS, (c) MDA, (d) 8-OHdG and (e) T-AOC content levels were test with ELISA. Data represent mean ± SD, *n* = 5. ^#^*P* < 0.05 versus the control group, ^*∗*^*P* < 0.05 versus the H_2_O_2_ group, ^△^*P* < 0.05 versus the morroniside 5 *μ*M group, ^□^*P* < 0.05 versus the morroniside 10 *μ*M group, and ^&^*P* < 0.05 versus the morroniside 20 *μ*M group. Scale bar = 200 *μ*m.

To assess the effect of morroniside on GC ROS levels induced by H_2_O_2_, we performed ROS fluorescence detection and ELISA. As shown in Figures 2(a) and 2(b), compared with the control group, ROS levels in GCs treated with 600 *μ*M H_2_O_2_ for 24 h were significantly increased (*P* < 0.05). Compared with the H_2_O_2_ group, ROS levels in morroniside-pretreated GCs were significantly decreased in a concentration-dependent manner, with peak effects at 20 *μ*M (*P* < 0.05). NAC is a potent antioxidant that can reduce the oxidative stress of GCs [24]. As shown in Figure 2(a), the ROS level in 1 mM NAC group was lower than that in 5 *μ*M morroniside group and higher than that in 20 *μ*M morroniside group (*P* < 0.05), but there was no significant difference from that in 10 *μ*M morroniside group (*P* > 0.05). The ROS level in 5 mM NAC group was significantly lower than that in 5, 10, and 20 *μ*M morroniside groups (*P* < 0.05).

MDA, 8-OHdG, and T-AOC levels were detected by ELISA to evaluate the degree of oxidative stress. The oxidative damage products content of MDA and 8-OHdG in the morroniside group was significantly reduced compared with the H_2_O_2_ group (*P* < 0.05), consistent with the ROS level in GCs (Figures 2(c) and 2(d)). The activity of T-AOC was detected to evaluate the antioxidant level of morroniside. The levels of T-AOC in GCs were significantly reduced after H_2_O_2_ treatment, whereas morroniside significantly increased the activities of T-AOC (*P* < 0.05) (Figure 2(e)). These results demonstrate that morroniside protects GCs by reducing oxidative damage induced by H_2_O_2_.

### 3.3. Morroniside Increases the Expression of SOD and NQO1 in GCs Inhibited by H_2_O_2_

**Figure 3:**
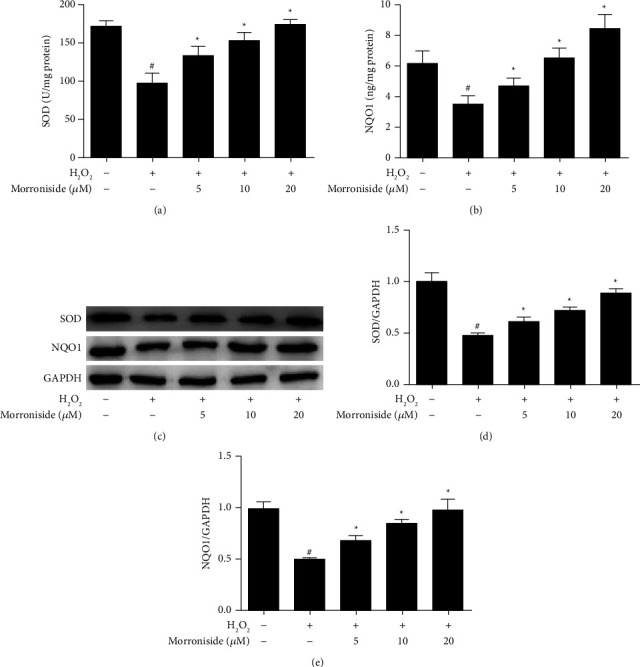
Effect of morroniside on the activity and protein expression of SOD and NQO1 in GCs inhibited by H_2_O_2_. The activity levels of antioxidant enzymes (a) SOD and (b) NQO1 were detected with ELISA. (c) The protein expression levels of SOD and NQO1 were detected by western blot analysis. (d) and (e) Densitometric analyses for western blots in (c). Data represent mean ± SD, *n* = 3. ^#^*P* < 0.05 versus the control group, ^*∗*^*P* < 0.05 versus the H_2_O_2_ group.

NQO1 is believed to partly reduce the free radical load in cells and the detoxification of xenobiotics. SOD is one of the most important antioxidant enzymes, enabling organisms to survive in an oxygen-containing atmosphere [25]. The levels of SOD and NQO1 in GCs were detected; H_2_O_2_ significantly reduced their contents, whereas morroniside significantly increased their contents (*P* < 0.05) (Figures 3(a) and 3(b)). The protein expression of SOD and NQO1 was also detected by Western blot analysis. The results showed that H_2_O_2_ reduced the protein levels of SOD and NQO1, while different concentrations of morroniside significantly increased the protein levels in a dose-dependent manner (Figures 3(c)–3(e)). These results demonstrate that morroniside upregulates the protein levels and contents of antioxidant enzymes to protect GCs against oxidative damage induced by H_2_O_2_.

### 3.4. Morroniside Inhibits GC Apoptosis Induced by Oxidative Stress

**Figure 4:**
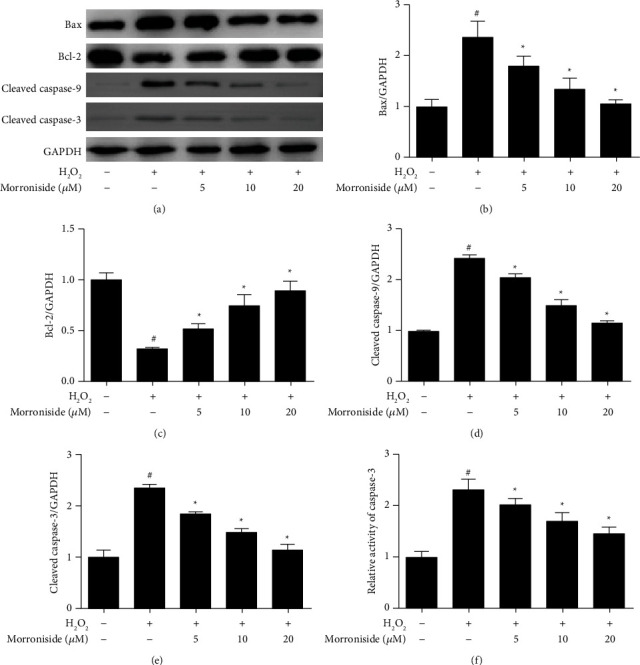
Effect of morroniside on GCs apoptosis induced by oxidative stress. (a) The protein expression levels of Bax, Bcl-2, cleaved caspase-9 and cleaved caspase-3, which were related to apoptosis, were detected by western blot analysis. (b–e) Densitometric analyses for Western blots in (a). (f) The casepase-3 activity level. Data represent mean ± SD, *n* = 3. ^#^*P* < 0.05 versus the control group, ^*∗*^*P* < 0.05 versus the H_2_O_2_ group.

High concentrations of ROS can damage the mitochondrial structure, causing the polar pores in the inner mitochondrial membrane to expand, which leads to an outflow of calcium ions and cytochrome C, finally causing the membrane potential to disappear and initiating apoptosis [26]. The expression of apoptosis-related proteins was detected by Western blot analysis. The results showed that the protein expressions of Bax, cleaved caspase-9, and cleaved caspase-3 in the H_2_O_2_ group were significantly increased, whereas Bcl-2 expression was significantly decreased (*P* < 0.05). However, compared with the H_2_O_2_ group, the protein expression levels of Bax, cleaved caspase-9, and cleaved caspase-3 were significantly decreased, whereas Bcl-2 levels were significantly increased after pretreatment with morroniside (Figures 4(a)–4(e)) (*P* < 0.05). H_2_O_2_ increased caspase-3 activity, whereas morroniside at different concentrations decreased its activity (Figure 4(f)) (*P* < 0.05). These results suggest that morroniside can attenuate the oxidative stress-induced apoptosis of GCs.

### 3.5. Morroniside Activates Nrf2 Signaling Pathways to Ameliorate Oxidative Stress in GCs

**Figure 5:**
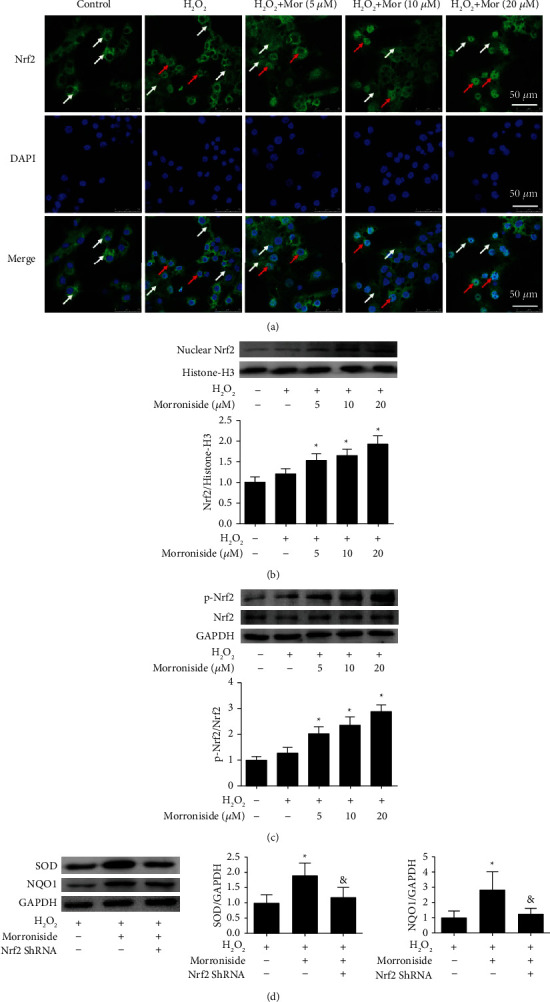
Effect of morroniside on the protein expression and nuclear translocation of Nrf2. (a) The green fluorescence represents Nrf2, and the nucleus is blue DAPI. The white arrows represent Nrf2 in the cytoplasm and the red arrows represent Nrf2 in the nucleus. Scale bars = 50 *μ*m. The protein expression levels of (b) nuclear Nrf2, (c) p-Nrf2, Nrf2 were detected by western blot analysis, and the protein expression levels of p-Nrf2 in nucleus, p-Nrf2/Nrf2 were quantitatively analyzed. (d) GCs were infected with sh-Nrf2, then treated with morroniside and H_2_O_2_. SOD and NQO1 expression were detected by western blot analysis. The concentration of morroniside in (d) was 10 *μ*M. Densitometry analysis of the western blots were shown at the right. Data represent mean ± SD, *n* = 3. ^*∗*^*P* < 0.05 versus the H_2_O_2_ group, and ^&^*P* < 0.05 versus the morroniside group.

Nrf2 normally remains in a low transcriptional state in the cytoplasm. When the cell is stimulated by ROS, Nrf2 is activated to p-Nrf2, which translocated to the nucleus where it combines with the ARE to activate the expression of antioxidant enzymes, thus having an important antioxidative effect [27]. To further study the mechanism underlying the antioxidant activity of morroniside, we assessed the effect of morroniside on the nuclear translocation of Nrf2 in GCs cultured *in vitro*. In the control and H_2_O_2_ group, Nrf2 was almost located in the cytoplasm. When GCs were pretreated with different concentrations morroniside, Nrf2 translocated to the nucleus, and cells treated with 20 *μ*M morroniside group were almost located in the nucleus (Figure 5(a)). Western blot analysis also showed that the level of Nrf2 in the nucleus in the morroniside group was significantly higher than that in the H_2_O_2_ group (*P* < 0.05) (Figure 5(b)), consistent with Figure 5(a). The levels of p-Nrf2 were detected by Western blot analysis. Compared with the H_2_O_2_ group, p-Nrf2 levels were increased in the different morroniside groups (*P* < 0.05) (Figure 5(c)). The results showed that morroniside induced p-Nrf2 expression and activated Nrf2 translocated into the nucleus to regulate the expression of antioxidant enzymes and thus exert antioxidant effects.

Then, we treated GCs with sh-Nrf2 virus to knock down Nrf2 expression and detected the SOD and NQO1 protein levels by Western blot analysis. The results showed that morroniside could induce SOD and NQO1 expression in GCs inhibited by H_2_O_2_. After the Nrf2 knockdown, the levels of SOD and NQO1 were significantly reduced even given morroniside (Figure 5(d)), which suggested that Nrf2 signaling pathway was an important way for morroniside to exert its antioxidant effect.

### 3.6. Morroniside Downregulates the p38 and JNK Signaling Pathways to Inhibit Apoptosis in GCs

**Figure 6:**
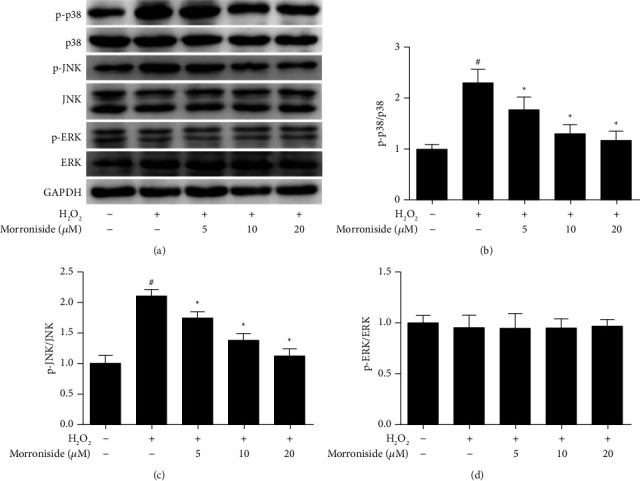
Effect of morroniside on the protein expression of MAPK signaling pathway. (a) The protein expression levels of p-p38, p38, p-JNK, JNK, p-ERK, and ERK were detected by western blot analysis. (b-d) Densitometric analyses for western blots in (a). Data represent mean ± SD, *n* = 3. ^#^*P* < 0.05 versus the control group, ^*∗*^*P* < 0.05 versus the H_2_O_2_ group.

MAPKs are activated in response to oxidative stress. Several studies have demonstrated that ROS production and activation of MAPKs play a vital role in *β*-amyloid-induced apoptosis [28]. To further elucidate the signaling pathway involved in the protective effects of morroniside against H_2_O_2_-induced apoptosis, we determined the effect of morroniside on MAPK activation in GCs. Our results showed that H_2_O_2_ upregulated p-JNK and p-p38 MAPK expression, but not p-ERK1/2 (Figure 6). Furthermore, morroniside significantly suppressed the H_2_O_2_-induced upregulation of p-JNK and p-p38 MAPK in GCs. These results suggested that morroniside can inhibit the JNK and p38 signaling pathways.

**Figure 7:**
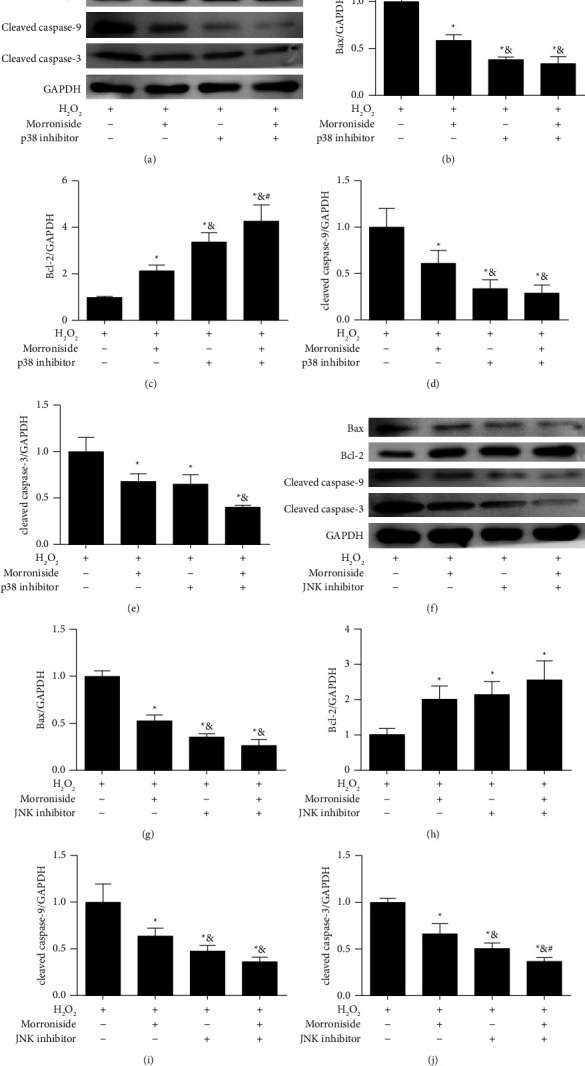
Morroniside downregulates the p38 and JNK signaling pathways to inhibit apoptosis in GCs. (a) GCs were incubated with p38 inhibitor SB203580, and then treated with morroniside and H_2_O_2_. The protein expression levels of Bax, Bcl-2, cleaved caspase-9, and cleaved caspase-3 were detected by western blot analysis. (b–e) Densitometric analyses for western blots in (a). (f) GCs were incubated with JNK inhibitor SP600125, and then treated with morroniside and H_2_O_2_. The protein expression levels of Bax, Bcl-2, cleaved caspase-9, and cleaved caspase-3were detected by western blot analysis. (g–j) Densitometric analyses for western blots in (f). The concentration of morroniside was 10 *μ*M. Data represent mean ± SD, *n* = 3. ^*∗*^*P* < 0.05 versus the H_2_O_2_ group, and ^&^*P* < 0.05 versus the morroniside group, ^#^*P* < 0.05 versus the H_2_O_2_ + p38 inhibitor group or the the H_2_O_2_ + JNK inhibitor group.

We also detected the effects of morroniside-inhibited p38MAPK and JNK pathway on apoptosis-related proteins by Western blot analysis. The results showed that compared with the H_2_O_2_ group, the protein expression levels of Bax, cleaved caspase-9, and cleaved caspase-3 were decreased, and Bcl-2 protein level was increased significantly in morroniside group (*P* < 0.05) (Figure 7). Similarly to the morroniside group, the addition of p38 (Figures7(a)–7(e)) and JNK (Figures 7(f)–7(j)) inhibitors also significantly decreased the protein expression of Bax, cleaved caspase-9, and cleaved caspase-3, and increased Bcl-2 protein expression in GCs treated with H_2_O_2_. In addition, morroniside combined with p38 or JNK inhibitors further induced more significant changes in the expression levels of apoptosis-related proteins. These results again confirmed that morroniside exerts an antiapoptotic effect by inhibiting the activation of p38 and JNK pathways.

## 4. Discussion

In this study, we first investigated the protective effects of morroniside on ovarian GCs from the perspective of oxidative stress. We showed that morroniside increased the expression level of p-Nrf2, promoted the nuclear translocation of Nrf2, upregulated the expression of antioxidant enzymes such as SOD and NQO1, and reduced the oxidative damage induced by H_2_O_2_. In addition, morroniside ameliorated p38 and JNK pathway-induced apoptosis by reducing ROS levels. Thus, morroniside can be used as a potential drug to improve the quality of follicles by protecting GCs.

Oxidative stress is closely related to the injury of female reproductive function. With increasing age, the antioxidant capacity of the ovary decreases, and the imbalance between oxidation and antioxidant in the ovary leads to the apoptosis of oocytes and ovarian GCs [29]. The level of ROS and expression of apoptotic proteins induced by ROS in the ovarian GCs of patients with PCOS were significantly higher than those of non-PCOS women [30]. Prieto et al. [31] confirmed that the levels of ROS in the follicular fluid of infertility patients with endometriosis are increased, while the levels of T-AOC and SOD are generally decreased. Morroniside suppresses autophagy and apoptosis in rat ovarian GCs through the PI3K/AKT/mTOR pathway [23]. Our study showed that morroniside inhibited the levels of ROS, 8-OHdG, and MDA and increased the expression of SOD and NQO1 in human ovarian GCs (Figures 2 and 3). The oxidative damage of GCs caused by various reasons such as aging directly affects female reproduction. The results of this study suggest that morroniside protects GCs from oxidative damage.

Nrf2 is important for antioxidant stress. When cells are subject to a variety of stimuli including antioxidants and xenobiotics, Nrf2 is activated and translocates into the nucleus, forms a complex with the MAF protein and binds to the ARE, and regulates ARE-mediated antioxidant enzyme gene expression such as SOD and NQO1 [12, 13]. The expression of Nrf2 detected in cumulus cells might be related to oocyte quality [32], whereas the upregulation of Nrf2 in oocytes and cumulus cells might affect the glutathione level in mature cumulus oocyte complexes [33]. Under the stimulation of harmful conditions (such as heat stress and heavy metals), the activation of the Nrf2 pathway can affect the activity and proliferation of ovarian GCs [34, 35]. However, Nrf2 activators such as quercetin and dimethyl fumarate [36] can upregulate the expression of Nrf2 and its downstream SOD and catalase in ovarian GCs to reduce the level of ROS, thus playing an antioxidant role [37]. Similar to these studies, our results showed that morroniside promoted the nuclear translocation of Nrf2, thereby regulating the expression of downstream antioxidant genes such as SOD and NQO1, thereby playing a protective role in GCs.

Endogenic ROS as a second messenger is involved in multiple signaling pathways of cascading effect [38] and is an upstream activator of p38 and JNK, which are the members of the MAPK family and are involved in the activation of apoptotic factors such as caspase-3 [30, 39, 40]. The release of cytochrome C is the key to the mitochondria-mediated activation of apoptosis protein, which activates caspase-3 together with its cofactor caspase-9 [41], and then activates the apoptotic signaling pathway. Bcl-2 and Bax are involved in this process. Bcl-2 inhibits the release of cyt C, while Bax promotes its release [42]. We obtained the same results showing that the morroniside reduced the phosphorylation levels of p38 and JNK; decreased the expression of Bax, cleaved caspase-9, and cleaved caspase-3; and increased the level of Bcl-2 in ovarian GCs. ERK also belongs to the MAPK family and is widely expressed in the GCs. Different from the apoptotic effects of p38 and JNK, ERK is very necessary for oocyte maturation and embryo development [43]. Han et al.[44] found that the p-ERK level in the GCs of women with low ovarian function was lower than that of women with normal ovarian function and confirmed that activation of ERK pathway could inhibit apoptosis and reduce the expression level of cleaved caspase-3. Our results showed that morroniside reduced H_2_O_2_-induced GCs apoptosis by regulating the p38 and JNK pathway but not the ERK pathway.

## 5. Conclusions

**Figure 8:**
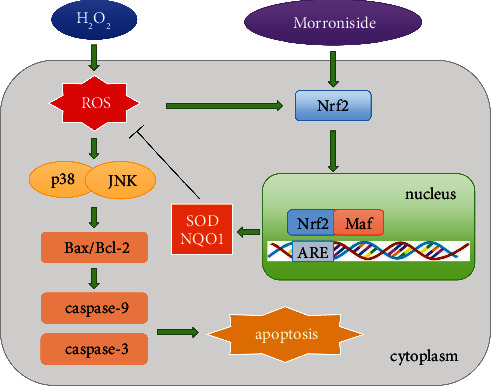
Schematic model of morroniside protecting human granulosa cells against H_2_O_2_-induced oxidative damage. Morroniside increased the expression level of p-Nrf2, promoted the nuclear translocation of Nrf2, and then upregulated the expression of antioxidant enzymes such as SOD and NQO1 which reduced the oxidative damage induced by H_2_O_2_. In addition, morroniside ameliorated p38 and JNK pathway-induced apoptosis by reducing ROS levels.

In summary, the results of our study suggested that the morroniside has a protective effect on GCs stimulated by H_2_O_2_. Morroniside increased the expression level of p-Nrf2, promoted the nuclear translocation of Nrf2, and upregulated the expression of antioxidant enzymes such as SOD and NQO1, which reduced the oxidative damage induced by H_2_O_2_. In addition, morroniside ameliorated p38 and JNK pathway-induced apoptosis by reducing ROS levels (Figure 8). This study also provides a new idea for the clinical treatment of reproductive diseases caused by oxidative stress.

## Data Availability

The data used and analyzed in this study are available from the corresponding author upon reasonable request.
